# Rice and beans: the nutritious, sustainable and affordable staple of the Brazilian diet

**DOI:** 10.1017/S1368980026102602

**Published:** 2026-04-30

**Authors:** Gabriela Lopes da Cruz, Kelly Garton, Boyd Swinburn, Maria Laura da Costa Louzada

**Affiliations:** 1 Department of Nutrition, School of Public Health, University of Sao Paulo, Brazil; 2 Center for Epidemiological Research in Nutrition and Health (NUPENS), University of Sao Paulo, Brazil; 3 Department of Epidemiology and Biostatistics, School of Population Health, University of Auckland, New Zealand

**Keywords:** Diet, Environment, Nutritional epidemiology, Brazil

## Abstract

**Objective:**

This study evaluated the consumption of rice and beans in Brazil, two staples of the Brazilian diet, by describing their consumption according to sociodemographic characteristics and assessing its association with nutritional quality, environmental impact and affordability of the diet.

**Design:**

Cross-sectional study.

**Setting:**

Brazil.

**Participants:**

We analysed food consumption data from 46 164 individuals aged 10 years and older, based on the most recent Household Budget Survey (2017–2018) in Brazil. The survey used a two-stage cluster sampling design and provides nationally representative data, covering all regions, states, metropolitan areas, capitals and urban and rural zones in Brazil.

**Results:**

In Brazil, rice and beans accounted for 10·75 % and 6·33 % of total daily energy intake, respectively. Their consumption was important across all sociodemographic groups analysed. Rice and beans intake was associated with higher nutritional quality, reduced environmental impact and lower diet costs. Higher combined consumption of rice and beans was associated with a 44·49 % reduction in nutritional inadequacies in the diet, a 17·64 % decrease in carbon footprint, a 21·05 % decrease in water footprint and a 38·03 % reduction in total diet cost, compared to lower consumption.

**Conclusions:**

Promoting increased consumption of rice and beans in Brazil offers a culturally appropriate solution in response to the global call for healthier and more sustainable diets, and is the most effective approach to improve human health and environmental sustainability in an affordable way in Brazil.

Diets are intrinsically connected to human health and environmental sustainability. Unhealthy diets are responsible for about 11 million premature deaths annually worldwide^(1)^, whereas food production accounts for a third of global greenhouse gas emissions^(2)^ and is a major cause of freshwater pollution, soil degradation and loss of biodiversity^(3)^.

Long-term established diets worldwide are being compromised by a globalised food system, marked by the displacement of traditional foods for ultra-processed foods^(4)^. This phenomenon, known as the nutrition transition, has led to an accelerated increase in diet-related diseases worldwide^(5)^ and calls for public health actions^(6)^.

While dietary improvements are vital for improving human health and reducing environmental impact, they present significant challenges, as most countries around the world are far from achieving their own national dietary guidelines recommendations – a situation further complicated by the affordability of diets that meet these recommendations^(7)^.

One important challenge is to find culturally appropriate solutions to address the increasing global demand for a transition to healthy and sustainable diets. To be effective, such shifts must align with local cultural heritage, values and preferences^(8)^. Understanding food combination patterns rather than focusing solely on individual food or nutrient intake enables a more comprehensive analysis of potential synergistic or antagonistic interactions within the diet.

Rice and beans figure largely among the most consumed foods in Brazil and are traditionally eaten together^(9)^. These two foods represent a staple of the Brazilian diet and serve as a symbol of the country’s food culture and culinary traditions. The consumption of rice and beans is emphasised as a dietary recommendation for promoting healthy eating according to the Dietary Guidelines for the Brazilian Population^(10)^, yet their consumption is decreasing^(11)^. The growing consumption of ultra-processed foods among the Brazilian population has displaced the intake of rice and beans over the past three decades^(11)^.

In the context of traditional diets, the Mediterranean diet has been extensively researched for its health benefits, while studies on traditional diets from non-European cultures remain limited^(12)^. Based on data from the most recent national food consumption survey, this study aimed to conduct an unprecedented evaluation of rice and beans consumption in Brazil, a staple of the Brazilian diet, by describing its consumption and assessing its association with nutritional quality, environmental impact and affordability of the diet.

## Methods

### Data source

We used data on individual food consumption from the most recent available Household Budget Survey (*Pesquisa de Orçamentos Familiares* – POF) conducted by the Brazilian Institute of Geography and Statistics – IBGE, from June 2017 to July 2018. The research employs a complex sampling design by clusters in two stages, involving the selection of census tracts followed by the listing of households. The census tracts were derived from the master sample of IBGE, grouped into strata of households with high geographical and socio-economic homogeneity^(13)^.

The research included 46 164 individuals aged 10 years and older, in a sample representative of the Brazilian population as a whole, the five macro-regions of the country, the twenty-seven federal units, the nine metropolitan regions, the twenty-seven state capitals and urban and rural areas.

### Sociodemographic characteristics

Socio-economic and demographic characteristics of the participants were collected using standardised questionnaires. In this study, we utilised the variables sex (male and female), age (adolescent – 10–19 years, adult – 20–59 years and elderly – 60 years or older), race/skin colour (*branco* (White), *negro (pretos e pardos)* (Black – including both Black and Brown individuals), *amarelo* (Asian) and *indigena* (Indigenous)), years of education (up to 4 years, 4–8 years, 8–12 years and more than 12 years), per capita household income (stratified into quintiles), area of residence (urban and rural) and macro-region of the country. We calculated per capita household income as the total household income divided by the number of residents.

### Individual food consumption

The evaluation of individual dietary intake was conducted using two 24-h dietary recalls on two non-consecutive days, based on the Automated Multiple-Pass Method^(14)^. Research agents collected information on all foods and beverages consumed by the participants on the day before the interview, including their quantities in household measures and preparation methods. The survey was carried out over four annual quarters to account for seasonal variations that occur over dietary consumption. Quantities considered improbable or missing were imputed using a similarity matrix based on variables correlated with the quantity consumed. A simple mean of the two recall days was calculated to estimate each participant’s average food consumption.

All culinary preparations (i.e. items made with more than one ingredient) reported in the survey were disaggregated using standardised recipes from the Brazilian Food Composition Table of the University of São Paulo, Food Research Center (FoRC), version 7.0, São Paulo, 2019. (Access available at: https://www.fcf.usp.br/tbca). We converted the consumed quantities of each food item into calories, carbohydrates, added sugars, protein, total fats, saturated fats, trans fats, dietary fibre, Na, potassium and Fe, using data from the same composition table. Energy density was calculated based on the solid fraction of the diet, by dividing the total caloric intake from solid foods by their corresponding weight in grams. We created the categories of rice and beans encompassing all reported varieties of minimally processed rice (parboiled, polished, long-grain, whole grain and wild) and beans (*carioca*, black, *fradinho* and green) in the survey.

### Assessment of the nutritional quality of the diet

The nutritional quality of the diet was assessed based on total dietary energy (calories/d), the percentage contribution of protein, carbohydrates, added sugar, total fats, saturated fats and trans fats to the total energy of the diet, the energy density of the diet (kcal/d) and the density (g or mg/1000 kcal) of fibre, Na, potassium and Fe. Within this set of nutrients and dietary components, the majority are linked to the risk of obesity and other chronic non-communicable diseases^(15)^ or are nutrients for which intake is insufficient among the Brazilian population^(13)^.

The number of inadequacies in the intake of nutrients considered markers of a healthy diet was calculated for each participant’s total diet. Using the cut-off points of the WHO^(15)^ for nutrients recognised as key markers of a healthy diet, inadequate intake of added sugars (≥ 10 % of total energy), total fats (≥ 30 % of total energy), saturated fats (≥ 10 % of total energy), trans fats (≥ 1 % of total energy), fibre (< 10 g/1000 kcal), Na (≥ 1 g/1000 kcal) and potassium (< 1755 mg/1000 kcal) was evaluated for each individual. An indicator was created to express the number of inadequacies in the consumption of nutrients, calculated from the simple sum, for each participant, of the number of nutrients with consumption outside the recommended limits (ranging, therefore, from 0 to 7).

### Assessment of environmental impact

The indicators used to assess environmental impacts were the carbon footprint and the water footprint of the diet. The carbon footprint is the measure of greenhouse gases, such as carbon dioxide, methane, nitrous oxide, hydrofluorocarbons, perfluorocarbons and sulfur hexafluoride, that are directly or indirectly generated by activities throughout the life cycle of a food^(16)^. This metric is represented in grams of carbon dioxide equivalent (gCO2eq). The water footprint indicates the total volume of freshwater consumed, also directly or indirectly, over the life cycle of a food. It includes the sum of surface water (blue water), rainwater (green water) and the water needed to absorb pollutants from production and consumption processes (grey water)^(17)^. The water footprint is expressed in litres.

We utilised data from the Footprints of Foods and Culinary Preparations Consumed in Brazil dataset^(18)^, which includes the environmental footprints of all foods mentioned in the POF 2017–2018. This dataset encompasses a cradle-to-retail scope, covering the entire life cycle of food from the farm to the point of sale, and considers the impacts of various cooking methods. The quantities of each food product reported in the survey were linked to the dataset to calculate the carbon and water footprints of each participant’s diet. The environmental impact was assessed by the carbon footprint (gCO2eq) and water footprint (L) of the diet per day.

### Assessment of affordability

The indicator used to assess affordability was the cost of the diet. We used data on food prices from the Household Food Supply module of the POF 2017–2018, the same survey that provided the food consumption data for this study. The Household Food Supply module collects information on the prices paid by households for each food item purchased for home consumption. Each household recorded both the quantity and price of every food item purchased, which we converted into prices per 100 g of edible portion. All prices were adjusted to a common reference date using official inflation rates provided by the survey data.

We grouped similar food items in 121 subgroups according to similarity (e.g. group of bananas, including different types of bananas such as *nanica*, *prata* and *ouro*; or group of vegetable oils, including soya oil, sunflower oil and maize oil), while also differentiating by degree of food processing when prices would show significant variation (e.g. instant noodles (ultra-processed foods) *v*. pasta (minimally processed foods)). The degree of food processing was defined according to the methodology used to apply the Nova food classification system to POF data^(19)^. Average prices were estimated at the subgroup level. All food items reported in the survey’s consumption module were organised into the same 121 subgroups, and the prices were linked accordingly.

For a more accurate estimation and to adjust for varying food prices across different locations in Brazil, price matching was conducted using the average price paid within the same household stratum for individuals residing in that stratum, accounting for 550 strata characterised by high geographical and socio-economic homogeneity.

The Household Food Supply module captures data only on food purchased for home consumption, while 24-h dietary recalls may include items not acquired for household use, resulting in the absence of price data in the strata. In such cases, the nationwide average price, calculated as the mean of reported prices across all strata, was applied. Affordability was evaluated based on the total price of the diet per day in Brazilian real (BRL).

### Data analysis

We assessed the association between socio-economic characteristics and the share of rice and beans in total dietary energy using crude linear regression analyses. We performed linear trend tests to assess the effect of age, years of education, and income as single continuous variables.

Nutritional, environmental and affordability indicators of the diet were calculated for Brazil as a whole and for quartiles of rice and beans intake in the diet. We analysed the combined effect of rice and beans on nutritional, environmental and affordability indicators using matrices that account for the combined consumption of rice and beans, based on quartiles of each group’s consumption relative to total dietary energy.

A joint analysis was conducted to create matrices reflecting the intersections of four quartiles of rice and four quartiles of beans contributions to total dietary energy intake. A joint variable with sixteen categories was generated to represent all possible combinations of quartiles of rice and beans consumption. To assess the nutritional impact, a matrix was created to evaluate the inadequate intake of nutrients considered markers of a healthy diet. The means of the number of inadequacies in nutrient consumption (which represents the sum of the number of nutrients with consumption outside the recommended limits, ranging from 0 to 7) were described according to quartiles of consumption of rice combined to quartiles of consumption of beans. Each nutrient was analysed individually using a separate matrix to compare its intake relative to the total diet, with results presented in the online Supplemental material.

The environmental impact was presented in two matrices indicating the carbon footprint and the water footprint of the total diet, and the affordability was presented using an indicator of the total price of the diet. Linear regression models were used to estimate the association between the combined categories of quartiles of rice and beans contribution to total dietary intake and the number of nutrient intake inadequacies, carbon and water footprints, and diet cost, using the category with the highest contribution of rice and beans (4th quartile on both) as a reference category.

Results were presented as crude values. The analysis was repeated, adjusting for the sociodemographic variables (sex, age, race/colour, income, years of education, area of residence and region) and presented in the online Supplemental material. Weighting factors were applied according to the sample structure, allowing the results to be extrapolated to the Brazilian population. Statistical significance was determined using a *p*-value threshold of ≤ 0·05. All analyses were performed using Stata statistical software, version 18.0.

This study complies with regulatory norms for research involving human beings in Brazil, as it uses data from a secondary source, made available by the IBGE, with guaranteed anonymity of participants.

## Results

In Brazil, the consumption of rice represented 10·75 % and beans 6·33 % of the total daily energy intake. Table 1 showed the contribution of rice and beans to total energy consumed in Brazil according to sociodemographic characteristics. The consumption of rice and beans was higher among males (+1·39 percentage points (pp) rice and +1·28 pp beans in relation to females), Black individuals (+1·87 pp rice and +1·47 pp beans in relation to White) and those living in rural areas (+2·03 pp rice and +1·59 pp beans in relation to urban). Across regions, the consumption of rice and beans was lowest in the South (8·43 % and 4·55 %, respectively) and highest in the Central-West (13·27 % and 7·18 %), with intermediate levels observed in the North, Northeast and Southeast. Rice and beans consumption was positively associated with age and inversely associated with years of education and income (*P* for linear trend ≤ 0·001) (Table 1).

**Table 1.** Percentage contribution of rice and beans to the total calories consumed by the Brazilian population aged 10 years and older, according to sociodemographic characteristics. POF, 2017–2018, Brazil

	Rice contribution to total dietary energy intake (%)					Beans contribution to total dietary energy intake (%)				
	Mean	CI 95 %	Coef. (*β*)	CI 95 %		Mean	CI 95 %	Coef. (*β*)	CI 95 %	
Brazil	10·75	10·64, 10·86				6·33	6·25, 6·41			
Sex										
Male	11·48	11·31, 11·64				7·00	6·88, 7·12			
Female	10·09	9·94, 10·24	−1·39	−1·57, −1·21	*	5·72	5·61, 5·82	−1·28	−1·41, −1·15	*
Age^†^										
Adolescent	10·48	10·22, 10·74				6·14	5·96, 6·33			
Adult	10·81	10·68, 10·95	0·33	0·02, 0·64		6·37	6·27, 6·47	0·23	0·01, 0·44	
Elderly	10·81	10·53, 11·10	0·33	−0·13, 0·78	***	6·37	6·19, 6·56	0·23	−0·08, 0·54	***
Race/colour										
White	9·70	9·54, 9·87				5·52	5·40, 5·64			
Black (Black and Brown)	11·57	11·42, 11·72	1·87	1·56, 2·17	*	6·99	6·88, 7·10	1·47	1·25, 1·69	*
Asian	11·24	9·09, 13·39	1·53	−0·66, 3·73		4·43	3·62, 5·24	−1·09	−1·98, −0·20	**
Indigenous	10·13	8·80, 11·46	0·43	−0·98, 1·84		5·46	3·66, 7·25	−0·06	−2·06, 1·93	
Years of education										
Up to 4 years	13·16	12·83, 13·49				8·05	7·82, 8·27			
4–8 years	11·79	11·57, 12·01	−1·37	−1·79, −0·95		7·13	6·98, 7·28	−0·92	−4·38, −3·55	
8–12 years	11·16	10·93, 11·39	−2·00	−2·44, −1·56		6·75	6·57, 6·92	−1·30	−1·61, −0·98	
More than 12 years	9·20	9·03, 9·36	−3·96	−4·38, −3·55	***	5·12	5·00, 5·24	−2·93	−3·24, −2·62	***
Quintiles of per capita family income										
1	13·28	13·02, 13·54				7·73	7·54, 7·92			
2	11·74	11·53, 11·95	−1·54	−2·07, −1·01		7·35	7·18, 7·52	−0·38	−0·78, 0·02	
3	10·89	10·62, 11·17	−2·39	−2·99, −1·78		6·65	6·47, 6·83	−1·08	−1·49, −0·67	
4	9·69	9·47, 9·91	−3·59	−4·13, −3·05		5·77	5·60, 5·94	−1·96	−2·36, −1·57	
5	8·16	7·93, 8·40	−5·12	−5·66, −4·57	***	4·16	4·00, 4·32	−3·57	−3·96, −3·19	***
Area of residence										
Urban	10·46	10·34, 10·58				6·10	6·01, 6·19			
Rural	12·49	12·26, 12·72	2·03	1·53, 2·53	*	7·69	7·52, 7·86	1·59	1·21, 1·97	*
Macro-region of the country										
North	10·96	10·59, 11·33				4·71	4·49, 4·93			
Northeast	10·95	10·78, 11·11	−0·01	−0·67, 0·64		6·40	6·29, 6·52	1·69	1·31, 2·07	*
Southeast	10·93	10·74, 11·13	−0·03	−0·69, 0·64		7·05	6·90, 7·20	2·34	1·93, 2·74	*
South	8·43	8·17, 8·68	−2·53	−3·23, −1·83	*	4·55	4·39, 4·70	−0·16	−0·57, 0·24	
Central-West	13·27	12·92, 13·62	2·31	1·48, 3·14	*	7·18	6·94, 7·41	2·47	1·98, 2·95	*

† Adolescent (10–17 years), adult (18–59 years) and elderly (60 years and older).

* *P* ≤ 0·005 compared to the reference category.

** *P* ≤ 0·05 compared to the reference category.

*** *P* for linear trend ≤ 0·001.

Table 2 presents the nutritional, environmental and affordability indicators of diets in Brazil overall and according to quartiles of rice and beans contribution to the diet. Among nutritional indicators, higher rice and beans intake was associated with lower total dietary energy and energy density and lower intake of added sugar, total fat, saturated fat and trans fat, while protein, carbohydrates, fibre, Na, potassium and Fe intake were higher (*P* for linear trend ≤ 0·001). For environmental indicators, diets with higher rice and beans consumption were associated with a lower carbon and water footprint (*P* for linear trend ≤ 0·001). In terms of affordability, the total cost of the diet decreased as the contribution of rice and beans in the diet increased (P for linear trend ≤ 0·001), ranging from BRL 9·83 to BRL 6·76 across the extreme quartiles of rice consumption and from BRL 9·25 to BRL 7·00 across the bean quartiles. These associations remained significant after adjusting for sociodemographic covariates (online Supplemental Material 1).

**Table 2.** Nutritional, environmental and affordability indicators of the diet among the Brazilian population aged 10 years and older, and according to quartile fractions of rice and beans contribution to total dietary energy intake. POF 2017–2018, Brazil

	Total food consumption (Brazil)	Quartiles of rice contribution to total dietary energy (%)					Quartiles of beans contribution to total dietary energy (%)				
		1°	2°	3°	4°		1°	2°	3°	4°	
**Nutritional indicator**											
Total energy (kcal/d)	1746·90	1887·36	1834·14	1716·97	1549·12	*	1713·86	1841·38	1792·54	1639·81	*
Contribution (%) to total energy from:											
Protein	18·34	17·61	17·99	18·72	19·04	*	17·67	17·92	18·46	19·32	*
Carbohydrates	54·04	52·37	53·72	53·88	56·18	*	52·36	52·93	54·34	56·52	*
Added sugar	10·09	12·05	11·47	9·71	7·11	*	11·26	11·22	10·14	7·72	*
Total fat	29·66	30·82	30·13	29·88	27·82	*	30·64	30·46	29·49	28·06	*
Saturated fat	9·44	10·45	9·74	9·31	8·26	*	10·33	9·92	9·26	8·25	*
Trans fat	0·67	0·78	0·73	0·67	0·52	*	0·75	0·73	0·67	0·55	*
Energy density (kcal/g)a1	1·62	1·76	1·65	1·58	1·48	*	1·79	1·68	1·58	1·42	*
Dietary fibre density (g/1000 kcal)	12·83	9·95	11·86	13·60	15·93	*	7·61	9·93	13·40	20·40	*
Na density (g/1000 kcal)	1·47	1·43	1·45	1·48	1·53	*	1·43	1·42	1·48	1·57	*
Potassium density (mg/1000 kcal)	1277·74	1230·29	1271·76	1301·64	1307·27	*	1161·07	1193·70	1282·12	1474·14	*
Fe density (mg/1000 kcal)	10·88	6·12	6·32	6·41	6·36	*	5·72	5·95	6·33	7·20	*
**Environmental indicator**											
Carbon footprint (gCO2eq/d)	4838·51	5124·31	5088·06	4795·98	4345·65	*	4840·72	5195·29	4952·24	4365·64	*
Water footprint (L/d)	4355·43	4710·62	4670·54	4294·07	3746·40	*	4311·22	4699·40	4490·89	3920·04	*
**Affordability indicator**											
Total cost of the diet (BRL/d)	8·43	9·83	8·99	8·14	6·76	*	9·25	9·14	8·32	7·00	*

Rice contribution (mean (CI 95 %)) to total dietary energy across quartiles of rice consumption: 1st quartile 2·57 (2·52, 2·63), 2nd quartile 7·26 (7·23, 7·30), 3rd quartile 11·65 (11·61, 11·70) and 4th quartile 21·53 (21·35, 21·71). Beans contribution to total dietary energy across quartiles of beans consumption: 1st quartile 3·06 (2·96, 3·17), 2nd quartile 5·32 (5·20, 5·44), 3rd quartile 7·31 (7·16, 7·46) and 4th quartile 9·63 (9·45, 9·82).

*P for linear trend ≤ 0·001.

Tables 3–6 illustrate matrices of the combined effects of rice and beans consumption on nutritional, environmental and affordability indicators. The number of nutritional inadequacies in the diet, as measured by key nutrients indicating a healthy diet (e.g. added sugar, total fat, saturated fat, trans fat, Na, potassium and fibre), decreased progressively from 4·54 in the lowest combined consumption quartiles to 2·52 in the highest combined consumption quartiles of rice and beans (Table 3), representing a 44·49 % reduction in the number of nutritional inadequacies in the diet. An overall improvement in diet quality was observed across all assessed nutrients, with the exception of Na (online Supplemental Material 2).

**Table 3.** Matrix of the number of inadequacies in nutrient intake^*^ of the diet according to quartiles of rice and bean contribution to total dietary energy. POF 2017–2018, Brazil

	Number of nutritional inadequacies in the diet							
	Quartiles of rice contribution to total dietary energy (%)							
	1		2		3		4	
Quartiles of beans contribution to total dietary energy (%)	Mean	se	Mean	se	Mean	se	Mean	se
1	4·54	0·02	4·45	0·03	4·33	0·03	3·90	0·03
2	4·53	0·03	4·44	0·03	4·32	0·03	3·89	0·03
3	3·92	0·03	3·83	0·02	3·71	0·02	3·28	0·02
4	3·16	0·03	3·07	0·02	2·96	0·02	2·52	0·02

The mean number of nutrient inadequacies observed in the highest performers on both quartiles of rice and beans contribution to total diet (4th quartile of rice and 4th quartile of beans) was significantly lower than the means for all other combined quartiles (*P* ≤ 0·001).

* Added sugar ≥ 10 % of total energy, total fats ≥ 30 % of total energy, saturated fat ≥ 10 % of total energy, trans fat ≥ 1 % of total energy, fibre < 12·5 g/1000kcal, Na ≥ 1 g/1000 kcal and potassium < 1755 mg/1000 kcal.

**Table 4.** Matrix of carbon footprints of the diet according to quartiles of rice and bean consumption. POF 2017–2018, Brazil

	Carbon footprint of the diet (gCO2eq/d)							
	Quartiles of rice contribution to total dietary energy (%)							
	1		2		3		4	
Quartiles of beans contribution to total dietary energy (%)	Mean	se	Mean	se	Mean	se	Mean	se
1	4984·22	67·99	4926·13	69·56	4699·19	65·57	4319·81	67·04
2	5388·54	71·11	5330·45	70·99	5103·50	62·88	4724·13	63·83
3	5219·22	68·44	5161·13	58·82	4934·19	57·28	4554·82	57·58
4	4769·44	71·62	4711·35	62·27	4484·41	53·16	4105·04	47·92

The mean carbon footprint observed in the highest performers on both quartiles of rice and beans contribution to total diet (4th quartile of rice and 4th quartile of beans) was significantly lower than the means for all other combined quartiles (*P* ≤ 0·001).

**Table 5.** Matrix of water footprints of the diet according to quartiles of rice and bean consumption. POF 2017–2018, Brazil

	Water footprint of the diet (l/d)							
	Quartiles of rice contribution to total dietary energy (%)							
	1		2		3		4	
Quartiles of beans contribution to total dietary energy (%)	Mean	se	Mean	se	Mean	se	Mean	se
1	4521·63	60·70	4422·90	62·20	4086·44	60·07	3592·19	60·19
2	4983·60	63·08	4884·87	59·54	4548·42	55·94	4054·16	56·36
3	4880·56	62·60	4781·83	54·99	4445·37	51·97	3951·12	53·77
4	4499·14	63·52	4400·42	56·40	4063·96	47·47	3569·70	41·92

The mean water footprint observed in the highest performers on both quartiles of rice and beans contribution to total diet (4th quartile of rice and 4th quartile of beans) was significantly lower than the means for all other combined quartiles (*P* ≤ 0·001).

**Table 6.** Matrix of total price of the diet according to quartiles of rice and bean consumption. POF 2017–2018, Brazil

	Total price of the diet (BRL/d)							
	Quartiles of rice contribution to total dietary energy (%)							
	1		2		3		4	
Quartiles of beans contribution to total dietary energy (%)	Mean	se	Mean	se	Mean	se	Mean	se
1	9·94	0·10	9·26	0·10	8·62	0·10	7·41	0·09
2	10·08	0·09	9·40	0·09	8·75	0·08	7·55	0·08
3	9·54	0·09	8·86	0·07	8·22	0·07	7·01	0·06
4	8·69	0·09	8·01	0·07	7·37	0·07	6·16	0·05

The mean price observed in the highest performers on both quartiles of rice and beans contribution to total diet (4th quartile of rice and 4th quartile of beans) was significantly lower than the means for all other combined quartiles (*P* ≤ 0·001).

Regarding environmental impact, both carbon and water footprints were lower among individuals with the highest combined consumption of rice and beans compared to those with the lowest. As presented in Table 4, the carbon footprint of the diet decreased from 4984·22 to 4105·04 gCO_2_eq, a 17·64 % reduction in emissions. The water footprint declined from 4521·63 to 3569·70 litres, representing 21·05 % less water use (Table 5).

Finally, the total cost of the diet declined as rice and beans consumption increased, from BRL 9·94 in the lowest combined consumption quartiles to BRL 6·16 in the highest, representing a 38·03 % reduction in total dietary cost (Table 6). Linear regression analyses showed that the mean number of nutrient inadequacies, carbon and water footprints, and total price of the diet observed in the highest performers on both quartiles of rice and beans (4th quartile of rice and 4th quartile of beans) was significantly lower than the means observed in all other combined quartiles (*P* ≤ 0·001). Results remained the same in the models adjusted for socio-economic variables (online Supplemental Material 3).

## Discussion

The consumption of rice and beans in Brazil is a local tradition associated with higher nutritional quality, lower environmental impact and reduced costs of the diet. This consumption was important across all sociodemographic groups evaluated in this study, ranging from 12·3 % to 21·2 % of daily energy intake, and was high across all regions of the country, reaching up to one-fifth of the dietary energy in the Central-West region. The consumption of rice and beans represented one-sixth of the total caloric intake of the population in 2017–2018.

Rice and beans is believed to have become a popular dish in Brazil in the 19th century, a time when rice began to replace maize and cassava flours in Brazilian cuisine. Beans, already a staple in the diet of Indigenous people in Brazil, were largely consumed mixed with flour. Rice, on the other hand, originates from Asia and was introduced to Brazil by the Portuguese^(20)^. Currently, Brazil is the largest rice producer and consumer outside Asia and ranks eleventh globally in production^(21)^.

Across different regions of the world, the combination of a cereal and a legume plays a key role in long-established diets. In addition to rice and beans, similar pairings include maize and beans in Latin America, wheat and chickpeas in the Middle East and North Africa, rice and lentils in South Asia, and rice and soybeans in East Asia^(22)^. The cereal–legume combination is present in the Mediterranean diet, the most extensively studied dietary pattern, known for its protective effects against chronic diseases and promoting longevity^(23)^. Cereals and legumes together may meet protein requirements for adults, with rice and beans complementing each other to provide all essential amino acids that must be obtained through the diet^(24)^, offering a high-value plant protein alternative to animal protein. In Brazil, protein intake meets recommended levels across all income groups^(25)^, with beans making an important contribution^(13)^. Rice and beans also ensure micronutrients intake and are the primary sources of dietary fibre in Brazil^(26)^.

This study demonstrated that rice and beans was associated with a more favourable dietary nutritional profile according to WHO recommendations for chronic disease prevention, including lower intake of added sugars, fats, saturated fats and trans fats, along with higher levels of dietary fibre, Fe and potassium. Na was the only nutrient for which intake did not improve towards adequacy. In Brazil, more than half of the population exceeds maximum recommended Na intake levels^(13)^, underscoring the importance of the dietary guidelines recommendation to limit the use of salt during cooking and seasoning^(10)^.

Diets with a higher intake of rice and beans had 45 % less nutritional inadequacy for critical nutrients compared to diets with lower intake. Studies show that the Brazilian dietary pattern based on rice and beans is linked to a lower risk of overweight and obesity, as well as healthier eating practices^(27,28)^. Bean consumption has been associated with protective effects against various diseases including diabetes, CVD, cancer and microbial infections^(29)^.

Dietary patterns high in rice and beans were associated with an 18 % lower carbon footprint and a 21 % lower water footprint compared to those with low consumption, suggesting that increasing the intake of rice and beans has the potential for reducing the environmental impact of current food systems. This is particularly relevant given beans’ agricultural ability to fix atmospheric nitrogen enriching both the plant and the soil, which decreases the need for synthetic fertilisers^(30)^. The cereal–legume combination aligns with dietary patterns rich in grains and plant-based proteins, which are recommended for both human and planetary health^(31)^. There has been an increase in greenhouse gas emissions and water use in the Brazilian diet from 1987 to 2018, alongside a decline in the consumption of unprocessed and minimally processed foods, among which rice and beans are important components^(32)^.

Dietary patterns with high rice and beans consumption had a total diet cost 38 % lower than those with low consumption. In Brazil, traditional healthy diets remain more affordable than those based on ultra-processed foods, mainly due to the low energy cost of staple grains like rice and beans^(33)^. Since the early 2000s, the price of ultra-processed foods has steadily decreased, becoming cheaper than processed foods and narrowing the price gap with unprocessed and minimally processed foods. Projections suggest that after 2025, unhealthy foods may cost less than healthy ones^(34)^.

This study analysed the diet’s affordability using 2017–2018 food price data, showing an association between diets with higher proportions of rice and beans and lower overall diet costs, with a cost of BRL 6·16 for diets with a greater contribution of rice and beans. However, costs of food in general are sensitive to inflationary fluctuations. For context, the price of the Basic Food Basket (per adult/d) in January 2025 was BRL 13·90, compared to BRL 7·83 in January 2018, based on the National Consumer Price Index^(35)^. With high nutritional value, rice and beans represent a valuable strategy to enhance dietary quality, address nutrient deficiencies and provide affordable, high-quality plant protein, particularly in regions with economic restrictions.

Traditional diets are centred around home-cooked meals made from biodiverse, locally available foods that were common before the widespread adoption of highly processed foods and industrial agriculture^(12)^. These diets reflect local food systems and culinary traditions that prioritise fresh, seasonal ingredients, usually associated with quality, safety and local tradition^(36,37)^. Across Asia, Latin America, Africa and the African diaspora, traditional diets tend to emphasise whole grains, dark green and orange vegetables, and legumes, all foods known for their health benefits^(12)^. Therefore, these traditions embody healthy eating patterns that should be recognised and supported, including through public policy initiatives.

Nutrition interventions and public health policies aligned with local culinary traditions are a more feasible approach to adopting healthy and sustainable eating habits. A study in China demonstrated that a culturally adapted diet facilitated a faster transition to sustainable and healthy eating compared to guidelines from the WHO, EAT-Lancet and Chinese Nutrition Society^(38)^. Similarly, a study with Mexican-heritage households in the USA showed that the most frequently purchased items accounted for fruits and vegetables of cultural significance to that population^(39)^, highlighting the potential benefits of incorporating culturally relevant foods into assistance programmes to improve nutrition and align with community preferences, enhancing the effectiveness of public health strategies.

Recent policies in Brazil align with this approach, including the implementation of the New Brazilian Food Basket in 2024, which emphasises a greater diversity of unprocessed and minimally processed foods, including regional varieties such as different types of beans consumed across the country, and the exclusion of ultra-processed foods^(40)^. Another key policy is the National School Feeding Program – PNAE, which, in 2024, incorporated foods such as *açaí* and *cupuaçu* into school meals in the North of the country, promoting regional dietary habits^(41)^. Focused policies and actions to promote increased rice and beans consumption in Brazil are yet necessary, such as ensuring affordable prices, expanding nutrition education, promoting their cultural significance and supporting sustainable production and distribution. These measures may enhance food and nutrition security, environmental sustainability and equitable access to food.

The strengths of this study include the probabilistic sampling method and national representativeness, based on data from over 30 000 individuals in urban and rural areas across the country. It also involved 2 d of dietary intake data collected using validated software, as well as a database with over 1200 food items. The nutritional composition table was specifically designed for the Brazilian population with rigorous quality control, and the environmental impact dataset was developed for foods within the Brazilian context. The estimates of food prices have limitations, as they are based on the average price paid within each household stratum rather than individual data and reflect retail prices. This means the methodology does not account for price variations in out-of-home meals, which could affect the overall estimates, although in this survey only 13 % of the population’s total energy intake came from food consumed outside home^(13)^. Nonetheless, these estimates represent the best available data, as the study relied on food price information from the same IBGE survey.

Other limitations relate to potential biases from dietary surveys, such as underreporting or overreporting of food consumption, discrepancies between actual and standardised recipes and differences between the actual nutritional composition of foods and the values provided in the nutritional composition table. To minimise these biases, data collection tools were pre-tested and validated, quality control procedures were implemented and inconsistent records were excluded or replaced with imputed values. This study used nutrient-based indicators to assess nutritional quality, with scope for future analyses to incorporate additional dimensions beyond nutrient adequacy, such as moderation, balance and variety, to further characterise traditional local dietary patterns.

Researching whole-food meal-based dietary patterns is essential for guiding dietary policies, nutrition education and sustainability initiatives rooted in local contexts, facilitating their implementation. In Brazil, the experience with dietary guidelines centred on locally prepared whole-food meals has been associated with important public health advances over a decade of implementation^(42)^. This approach is especially relevant where whole-food meal-based diets are still preserved, mostly low- and middle-income countries. Further research in this area is key to tackling the major public health challenge of the displacement of long-established dietary patterns by ultra-processed foods, a key driver of the global burden of multiple diet-related chronic diseases^(43)^.

## Conclusion

Dietary shifts are crucial for improving human health and reducing the environmental impact of food systems, and must be aligned with economic affordability. Encouraging increased consumption of rice and beans in Brazil offers a culturally appropriate solution in response to the global call for healthier and more sustainable diets. The focus on strengthening the Brazilian traditional diet is the most effective approach to improve human health and environmental sustainability in an affordable way in Brazil.

## Supporting information

10.1017/S1368980026102602.sm001 da Cruz et al. supplementary material 1 da Cruz et al. supplementary material

10.1017/S1368980026102602.sm002 da Cruz et al. supplementary material 2 da Cruz et al. supplementary material

10.1017/S1368980026102602.sm003 da Cruz et al. supplementary material 3 da Cruz et al. supplementary material

## Supplementary material

For supplementary material accompanying this paper visit https://doi.org/10.1017/S1368980026102602
