# Supplementary material for: Rice and beans: the nutritious, sustainable and affordable staple of the Brazilian diet
Source: Public Health Nutr. 2026 Apr 30;29(1):e114. doi: 10.1017/S1368980026102602 (PMC13434487; doi:10.1017/S1368980026102602)
Supplement: Supplementary file 1 [file S1368980026102602sup001.docx]

| **Supplememtary material 3.** Matrices of inadequacies in nutrient intake^a^, environmental impact (carbon and water footprint) and total diet cost, according to quartiles of rice and beans contribution to total dietary energy intake, adjusted for sex, age, race/skin colour, years of education, per capita household income, area of residence, and macro-region of the country. POF 2017-2018, Brazil. | | | | | | | | | | |
| --- | --- | --- | --- | --- | --- | --- | --- | --- | --- | --- |
|  | **Number of nutritional inadequacies in the diet (total)** | | | |  |  | **Total price of the diet (BRL/d)** | | | |
|  | Quartiles of rice contribution to total dietary energy (%) | | | |  |  | Quartiles of rice contribution to total dietary energy (%) | | | |
|  | 1 | 2 | 3 | 4 |  |  | 1 | 2 | 3 | 4 |
| Quartiles of beans contribution to total dietary energy (%) | Mean (SE) | Mean (SE) | Mean (SE) | Mean (SE) |  | Quartiles of beans contribution to total dietary energy (%) | Mean (SE) | Mean (SE) | Mean (SE) | Mean (SE) |
| 1 | 4.54(0.02) | 4.44(0.03) | 4.33(0.03) | 3.93(0.03) |  | 1 | 9.88(0.10) | 9.21(0.09) | 8.65(0.09) | 7.66(0.08) |
| 2 | 4.53(0.03) | 4.43(0.03) | 4.31(0.03) | 3.91(0.03) |  | 2 | 10.00(0.09) | 9.33(0.08) | 8.77(0.07) | 7.78(0.07) |
| 3 | 3.92(0.03) | 3.82(0.02) | 3.70(0.02) | 3.30(0.02) |  | 3 | 9.44(0.09) | 8.76(0.07) | 8.20(0.07) | 7.22(0.06) |
| 4 | 3.15(0.03) | 3.06(0.02) | 2.94(0.02) | 2.54(0.02) |  | 4 | 8.52(0.09) | 7.85(0.07) | 7.29(0.06) | 6.30(0.05) |
|  |  |  |  |  |  |  |  |  |  |  |
|  | **Carbon footprint of the diet (gCO2eq/d)** | | | |  |  | **Water footprint of the diet (l/d)** | | | |
|  | Quartiles of rice contribution to total dietary energy (%) | | | |  |  | Quartiles of rice contribution to total dietary energy (%) | | | |
|  | 1 | 2 | 3 | 4 |  |  | 1 | 2 | 3 | 4 |
| Quartiles of beans contribution to total dietary energy (%) | Mean (SE) | Mean (SE) | Mean (SE) | Mean (SE) |  | Quartiles of beans contribution to total dietary energy (%) | Mean (SE) | Mean (SE) | Mean (SE) | Mean (SE) |
| 1 | 4988.08(66.41) | 4946.74(66.74) | 4711.59(63.38) | 4267.04(66.97) |  | 1 | 4492.33(58.93) | 4447.54(60.01) | 4139.63(57.42) | 3609.26(60.93) |
| 2 | 5383.55(67.66) | 5342.22(67.94) | 5107.06(60.60) | 4662.51(63.17) |  | 2 | 4934.49(60.31) | 4889.70(57.14) | 4581.80(54.02) | 4051.43(57.81) |
| 3 | 5226.05(66.55) | 5184.71(56.76) | 4949.56(55.70) | 4505.01(56.66) |  | 3 | 4829.27(59.92) | 4784.48(52.72) | 4476.58(49.75) | 3946.20(53.57) |
| 4 | 4790.79(70.18) | 4749.46(60.33) | 4514.31(51.17) | 4069.76(46.63) |  | 4 | 4442.58(62.16) | 4397.79(55.04) | 4089.89(45.68) | 3559.51(41.21) |
|  |  |  |  |  |  |  |  |  |  |  |
| SE: standard error. | | | | | | | | | | |
| Note: The mean number of nutrient inadequacies, total price of the diet and carbon and water footprints observed in the highest performers on both quartiles of rice and beans contribution to total diet (4th quartile of rice and 4th quartile of beans) was significantly lower than the means for all other combined quartiles (p < 0.001). | | | | | | | | | | |
| ᵃ: Added sugar ≥ 10% of total energy, total fats ≥ 30% of total energy, saturated fat ≥ 10% of total energy, trans fat ≥ 1% of total energy, fibre < 12.5g/1000kcal, sodium ≥ 1 g/1,000 kcal, and potassium < 1,755 mg/1,000 kcal. | | | | | | | | | | |
