# Supplementary material for: Rice and beans: the nutritious, sustainable and affordable staple of the Brazilian diet
Source: Public Health Nutr. 2026 Apr 30;29(1):e114. doi: 10.1017/S1368980026102602 (PMC13434487; doi:10.1017/S1368980026102602)
Supplement: Supplementary file 2 [file S1368980026102602sup002.docx]

| **Supplementary Material 2**. Matrix of total dietary nutriet intake (added sugars, protein, total fats, saturated fats, trans fats, dietary fibre, sodium, potassium, and iron) according to quartiles of rice and beans contribution to total dietary energy intake. POF 2017-2018, Brazil. | | | | | | | | | | | | | | | | |
| --- | --- | --- | --- | --- | --- | --- | --- | --- | --- | --- | --- | --- | --- | --- | --- | --- |
|  | **Added sugar (%)** | | | |  |  | **Total fat (%)** | | | |  |  | **Trans fat (%)** | | | |
|  | Quartiles of rice contribution to total dietary energy (%) | | | |  |  | Quartiles of rice contribution to total dietary energy (%) | | | |  |  | Quartiles of rice contribution to total dietary energy (%) | | | |
|  | 1 | 2 | 3 | 4 |  |  | 1 | 2 | 3 | 4 |  |  | 1 | 2 | 3 | 4 |
| Quartiles of beans contribution to total dietary energy (%) |  |  |  |  |  | Quartiles of beans contribution to total dietary energy (%) |  |  |  |  |  | Quartiles of beans contribution to total dietary energy (%) |  |  |  |  |
| 1 | 12.16(0.15) | 11.80(0.16) | 10.35(0.15) | 8.04(0.15) |  | 1 | 31.08(0.15) | 30.67(0.14) | 30.69(0.15) | 28.85(0.15) |  | 1 | 0.80(0.01) | 0.77(0.01) | 0.72(0.01) | 0.59(0.01) |
| 2 | 12.43(0.15) | 12.07(0.14) | 10.63(0.14) | 8.31(0.14) |  | 2 | 31.05(0.14) | 30.64(0.13) | 30.66(0.14) | 28.82(0.14) |  | 2 | 0.79(0.01) | 0.76(0.01) | 0.71(0.01) | 0.58(0.01) |
| 3 | 11.80(0.15) | 11.44(0.13) | 10.00(0.13) | 7.68(0.13) |  | 3 | 30.26(0.14) | 29.86(0.12) | 29.88(0.12) | 28.04(0.12) |  | 3 | 0.75(0.01) | 0.72(0.01) | 0.68(0.01) | 0.55(0.01) |
| 4 | 10.23(0.15) | 9.87(0.14) | 8.43(0.12) | 6.11(0.11) |  | 4 | 29.28(0.15) | 28.87(0.13) | 28.89(0.12) | 27.05(0.10) |  | 4 | 0.67(0.01) | 0.64(0.01) | 0.59(0.01) | 0.46(0.01) |
|  |  |  |  |  |  |  |  |  |  |  |  |  |  |  |  |  |
|  | **Protein (%)** | | | |  |  | **Saturated fat (%)** | | | |  |  | **Dietary fibre (g/1,000 kcal)** | | | |
|  | Quartiles of rice contribution to total dietary energy (%) | | | |  |  | Quartiles of rice contribution to total dietary energy (%) | | | |  |  | Quartiles of rice contribution to total dietary energy (%) | | | |
|  | 1 | 2 | 3 | 4 |  |  | 1 | 2 | 3 | 4 |  |  | 1 | 2 | 3 | 4 |
| Quartiles of beans contribution to total dietary energy (%) |  |  |  |  |  | Quartiles of beans contribution to total dietary energy (%) |  |  |  |  |  | Quartiles of beans contribution to total dietary energy (%) |  |  |  |  |
| 1 | 17.40(0.11) | 17.58(0.11) | 18.12(0.12) | 18.30(0.12) |  | 1 | 10.74(0.06) | 10.29(0.06) | 10.06(0.07) | 9.16(0.06) |  | 1 | 7.56(0.06) | 7.41(0.07) | 7.43(0.07) | 8.38(0.08) |
| 2 | 17.55(0.14) | 17.73(0.10) | 18.28(0.11) | 18.46(0.11) |  | 2 | 10.48(0.06) | 10.03(0.06) | 9.80(0.07) | 8.90(0.06) |  | 2 | 9.87(0.06) | 9.72(0.06) | 9.74(0.06) | 10.69(0.07) |
| 3 | 17.96(0.12) | 18.14(0.10) | 18.69(0.11) | 18.87(0.10) |  | 3 | 9.98(0.06) | 9.53(0.05) | 9.30(0.06) | 8.40(0.05) |  | 3 | 13.28(0.07) | 13.13(0.06) | 13.15(0.05) | 14.10(0.06) |
| 4 | 18.65(0.13) | 18.82(0.10) | 19.37(0.09) | 19.55(0.08) |  | 4 | 9.27(0.06) | 8.81(0.05) | 8.59(0.05) | 7.69(0.04) |  | 4 | 20.09(0.09) | 19.93(0.08) | 19.95(0.08) | 20.90(0.08) |
|  |  |  |  |  |  |  |  |  |  |  |  |  |  |  |  |  |
|  | **Sodium (g/1,000 kcal)** | | | |  |  | **Potassium (mg/1,000 kcal)** | | | |  |  | **Iron (mg/1,000 kcal)** | | | |
|  | Quartiles of rice contribution to total dietary energy (%) | | | |  |  | Quartiles of rice contribution to total dietary energy (%) | | | |  |  | Quartiles of rice contribution to total dietary energy (%) | | | |
|  | 1 | 2 | 3 | 4 |  |  | 1 | 2 | 3 | 4 |  |  | 1 | 2 | 3 | 4 |
| Quartiles of beans contribution to total dietary energy (%) |  |  |  |  |  | Quartiles of beans contribution to total dietary energy (%) |  |  |  |  |  | Quartiles of beans contribution to total dietary energy (%) |  |  |  |  |
| 1 | 1.42(0.01) | 1.42(0.01) | 1.43(0.01) | 1.46(0.01) |  | 1 | 1176.27(7.18) | 1165.42(6.95) | 1147.15(7.25) | 1112.81(7.44) |  | 1 | 5.83(0.04) | 5.76(0.04) | 5.63(0.04) | 5.39(0.04) |
| 2 | 1.41(0.01) | 1.41(0.01) | 1.42(0.01) | 1.45(0.01) |  | 2 | 1214.31(6.84) | 1203.47(6.54) | 1185.19(6.37) | 1150.86(6.49) |  | 2 | 6.09(0.03) | 6.02(0.03) | 5.89(0.03) | 5.65(0.03) |
| 3 | 1.46(0.01) | 1.47(0.01) | 1.47(0.01) | 1.51(0.01) |  | 3 | 1309.69(6.96) | 1298.84(5.72) | 1280.57(5.60) | 1246.23(5.58) |  | 3 | 6.51(0.03) | 6.45(0.03) | 6.32(0.03) | 6.08(0.03) |
| 4 | 1.54(0.01) | 1.54(0.01) | 1.55(0.01) | 1.59(0.01) |  | 4 | 1514.18(7.62) | 1503.34(6.28) | 1485.07(5.47) | 1450.73(5.17) |  | 4 | 7.48(0.04) | 7.41(0.03) | 7.28(0.03) | 7.04(0.03) |
|  |  |  |  |  |  |  |  |  |  |  |  |  |  |  |  |  |
| SE: standard error. | | | | | | | | | | | | | | | | |
