# Supplementary material for: Rice and beans: the nutritious, sustainable and affordable staple of the Brazilian diet
Source: Public Health Nutr. 2026 Apr 30;29(1):e114. doi: 10.1017/S1368980026102602 (PMC13434487; doi:10.1017/S1368980026102602)
Supplement: Supplementary file 3 [file S1368980026102602sup003.docx]

| **Supplementary Material 1.** Nutritional, environmental, and affordability indicators of the diet accoding to quartile fractions of rice and beans contribution to total dietary energy intake, adjusted for sex, age, race/skin colour, years of education, per capita household income, area of residence, and macro-region of the country. POF 2017-2018, Brazil. | | | | | | | | | | | | | |
| --- | --- | --- | --- | --- | --- | --- | --- | --- | --- | --- | --- | --- | --- |
|  | | |  | Quartiles of rice contribution to total dietary energy (%) | | | | | Quartiles of beans contribution to total dietary energy (%) | | | | |
|  | | |  | 1° | 2° | 3° | 4° |  | 1° | 2° | 3° | 4° |  |
| **Nutritional Indicator** | | |  |  |  |  |  |  |  |  |  |  |  |
|  | | Total energy (kcal/day) | | 1895.43 | 1837.99 | 1716.54 | 1536.63 | * | 1714.78 | 1836.31 | 1794.08 | 1641.36 | * |
| Contribution (%) to total energy from: | | |  |  |  |  |  |  |  |  |  |  |  |
|  | | Protein | | 17.68 | 18.04 | 18.71 | 18.93 | * | 17.66 | 17.95 | 18.46 | 19.29 | * |
|  | | Carbohydrates | | 52.46 | 53.80 | 53.91 | 55.98 | * | 52.55 | 52.92 | 54.29 | 56.39 | * |
|  | | Added sugar | | 11.96 | 11.40 | 9.73 | 7.26 | * | 11.23 | 11.14 | 10.15 | 7.81 | * |
|  | | Total fat | | 30.71 | 30.03 | 29.83 | 28.08 | * | 30.40 | 30.43 | 29.56 | 28.26 | * |
|  | | Saturated fat | | 10.32 | 9.68 | 9.33 | 8.43 | * | 10.19 | 9.87 | 9.29 | 8.41 | * |
|  | | Trans fat | | 0.78 | 0.73 | 0.67 | 0.53 | * | 0.75 | 0.72 | 0.67 | 0.55 | * |
| Energy density (kcal/g)a1 | | | | 1.76 | 1.65 | 1.58 | 1.48 | * | 1.80 | 1.68 | 1.58 | 1.41 | * |
| Fibre density (g/1,000 kcal) | | | | 10.35 | 11.99 | 13.48 | 15.51 | * | 7.58 | 9.92 | 13.40 | 20.42 | * |
| Sodium density (g/1,000 kcal) | | | | 1.44 | 1.45 | 1.48 | 1.53 | * | 1.43 | 1.42 | 1.48 | 1.56 | * |
| Potassium density (mg/1,000 kcal) | | | | 1228.33 | 1270.86 | 1300.74 | 1310.69 | * | 1139.64 | 1192.13 | 1287.90 | 1490.97 | * |
| Iron (mg/1,000 kcal) | | | | 6.19 | 6.33 | 6.38 | 6.30 | ** | 5.72 | 5.95 | 6.33 | 7.20 | * |
|  | | |  |  |  |  |  |  |  |  |  |  |  |
| **Environmental Indicator** | | | |  |  |  |  |  |  |  |  |  |  |
|  | Carbon Footprint (gCO2eq/day) | | | 5102.82 | 5105.87 | 4822.40 | 4318.64 | * | 4775.02 | 5176.73 | 4973.60 | 4424.32 | * |
|  | Water Footprint (L/day) | | | 4636.07 | 4676.84 | 4342.03 | 3763.26 | * | 4191.70 | 4681.09 | 4540.68 | 4004.72 | * |
|  | | |  |  |  |  |  |  |  |  |  |  |  |
| **Affordability indicator** | | |  |  |  |  |  |  |  |  |  |  |  |
|  | Total cost of the diet (BRL/day) | | | 9.65 | 8.87 | 8.15 | 7.04 | * | 9.07 | 9.04 | 8.37 | 7.23 | * |
|  | | |  |  |  |  |  |  |  |  |  |  |  |
| Adjusted values for sex, age, race/color, income, education, area, and region. | | | | | | | | |  |  |  |  |  |
| *: p for linear trend ≤ 0.001 | | | | |  |  |  |  |  |  |  |  |  |
| **: p for linear trend ≤ 0.05 | | | | |  |  |  |  |  |  |  |  |  |
| a World Health Organization (WHO). *Diet, Nutrition and the Prevention of Chronic Diseases*. Geneva: World Health Organization; 2003. | | | | | | | | | | | | |  |
| b The recommended value is based on a diet of 2,000 kcal. | | | | | | | |  |  |  |  |  |  |
| c World Health Organization (WHO). *World Health Organization Issues New Guidance on Dietary Salt and Potassium*. Geneva; 2013. The recommended value is based on a diet of 2,000 kcal. | | | | | | | | | | | | | |
